# Three cases of ARDS: An emerging complication of *Plasmodium vivax* malaria - Queries?

**DOI:** 10.4103/0970-2113.76313

**Published:** 2011

**Authors:** Syed Ahmed Zaki

**Affiliations:** *Department of Pediatrics, Lokmanya Tilak Municipal General Hospital, Sion, Mumbai 400 022, India E-mail: drzakisyed@gmail.com*

Sir,

We read with interest the recent case report on ‘Three cases of ARDS: An emerging complication of Plasmodium vivax malaria’ by Sarkar *et al*.,[1] and have the following comments to offer:

The third patient (15-year old boy) described in the case presented with progressive respiratory distress, and thus had severe manifestations of vivax malaria as per World Health Organisation (WHO) guidelines for the treatment of malaria.[2] As per the recommendations by WHO and Indian Academy of Pediatrics (IAP) for the treatment of severe malaria quinine, artesunate, or artemether should have been started.[2,3] Although this patient improved with chloroquine, it may not be the same for other patients with severe malaria. Treatment regimes for *uncomplicated* malaria can be tailored specifically according to the resistance pattern of the region where the patient resides. However, the same is not applicable in cases of *severe and complicated* malaria where the main objective of treatment is to prevent death. Prevention of recrudescence, transmission, or emergence of resistance and prevention of disabilities are of secondary importance. Severe malaria if left untreated has a mortality of 100%, and death often occurs within hours of admission. One cannot start chloroquine initially and then change to second line antimalarials in case the patient is not responding. Through this letter, I wish to re-emphasize to our readers that drug policy in all cases of severe malaria should be either intravenous quinine or parenteral artemisinin derivatives irrespective of chloroquine-resistance status.

## References

[1] Sarkar S, Saha K, Das CS (2010). Three cases of ARDS: An emerging complication of *Plasmodium vivax* malaria. Lung India.

[2] (2010). WHO. Guidelines for the treatment of malaria.

[3] Kundu R, Ganguly N, Ghosh TK (2008). Infectious Diseases Chapter, Indian Academy of Pediatrics. Management of malaria in children: Update 2008. Indian Pediatr.

